# Predicting the Incidence of Pressure Ulcers in the Intensive Care Unit Using Machine Learning

**DOI:** 10.5334/egems.307

**Published:** 2019-09-05

**Authors:** Eric M. Cramer, Martin G. Seneviratne, Husham Sharifi, Alp Ozturk, Tina Hernandez-Boussard

**Affiliations:** 1 Department of Biomedical Informatics, Stanford University, US; 2 Department of Computer Science, Stanford University, US

**Keywords:** electronic health records, machine learning, predictive analytics, pressure injury, intensive care

## Abstract

**Background:**

Reducing hospital-acquired pressure ulcers (PUs) in intensive care units (ICUs) has emerged as an important quality metric for health systems internationally. Limited work has been done to characterize the profile of PUs in the ICU using observational data from the electronic health record (EHR). Consequently, there are limited EHR-based prognostic tools for determining a patient’s risk of PU development, with most institutions relying on nurse-calculated risk scores such as the Braden score to identify high-risk patients.

**Methods and Results:**

Using EHR data from 50,851 admissions in a tertiary ICU (MIMIC-III), we show that the prevalence of PUs at stage 2 or above is 7.8 percent. For the 1,690 admissions where a PU was recorded on day 2 or beyond, we evaluated the prognostic value of the Braden score measured within the first 24 hours. A high-risk Braden score (<=12) had precision 0.09 and recall 0.50 for the future development of a PU. We trained a range of machine learning algorithms using demographic parameters, diagnosis codes, laboratory values and vitals available from the EHR within the first 24 hours. A weighted linear regression model showed precision 0.09 and recall 0.71 for future PU development. Classifier performance was not improved by integrating Braden score elements into the model.

**Conclusion:**

We demonstrate that an EHR-based model can outperform the Braden score as a screening tool for PUs. This may be a useful tool for automatic risk stratification early in an admission, helping to guide quality protocols in the ICU, including the allocation and timing of prophylactic interventions.

## Introduction

Pressure ulcers (PUs) represent a significant public health issue, afflicting intensive care units (ICUs) internationally [1]. PUs occur when the skin is exposed to pressure and shear, typically as a result of long term patient immobilization, causing injury to the epidermis and underlying tissues. The prevalence of PUs in ICUs has been estimated between 22–49 percent [2,3]. An ulcer can significantly extend a patients’ length of stay in the hospital, and can cause long term dis- ability, with muscles and other deep tissues often impaired for months after the resolution of the external wound [4]. PUs cause chronic morbidity through pain and associated psychological impacts; however, they are also responsible for significant mortality, typically as a consequence of sepsis following bacterial inoculation to the bloodstream via the ulcer.

PUs are, however, eminently preventable and treatable in their early stages. Consequently, the reduction of PUs in acute care has been identified by the National Quality Forum (NQF) and the Agency for Healthcare Research and Quality (AHRQ) as an important quality metric, and both agencies have published frameworks for tackling this issue [5,6]. Prophylactic measures for PUs include regular patient rolling, specialized pressure mattresses (e.g. powered active air and hybrid air surfaces), and good patient nutrition [7,8]. Management of established ulcers includes pressure dressings (hydrocolloid, foam and film), wound cleansers, negative pressure therapy, ultrasound therapy, and surgical intervention [9]. To reduce PU incidence, it is critical to identify at-risk patients and intervene early. As many of these therapies are labor-intensive or expensive (e.g. pressure mattresses), allocating resources according to patient risk is an important clinical challenge.

Several risk stratification methods have been developed, including the Norton (1965), Waterlow (1985), and Braden (proposed in 1987) scales. These are nurse-reported scores that combine local skin factors (such as moisture, friction and shear) with patient-level factors (such as mobility, sensory perception and nutrition) [10]. The sensitivity and specificity of these risk scores in predicting the later development of PUs is highly variable [11]. For example, a survey of 7,800 ICU patients found that the best performance of the Braden score yielded an area-under-the-curve (AUC) of only 0.67 [12]. One Brazilian study found that although the Braden score had poor prognostic accuracy, its performance could be increased by including a broader range of patient-level factors [13]. These variables included: age, gender, comorbidities (specifically diabetes, hematological malignancies, and peripheral artery disease), hypotension, renal replacement therapy and mechanical ventilation within the first 24 hours of admission. Most of these data are recorded in the EHR, raising the possibility to build more powerful predictive models using a broader range of variables than the traditional manual risk scores. This aligns with a recent meta-analysis of 17 studies evaluating these scores, which called for the development of more personalized risk algorithms [14].

There have been several early attempts to model the incidence of PUs with statistical methods. Kaewprag et al. used Bayesian nets to classify patients based on the presence of an ulcer, achieving good specificity but poor sensitivity, consistently below 0.5 [15]. Park et al. performed multivariate linear regression on 61 clinical and laboratory variables to predict time-to-ulcer onset in 202 patients with PUs [16]. Additionally, Cho et al. developed a Bayesian net to predict PUs based on 37 structured elements derived from the EHR [17]. Most of these algorithms do not deal well with time-dependence – focusing more on classifying PU versus non-PU patients for the purpose of identifying risk factors, rather than predicting ulcer development after an index time. The exception is Cho et al. – the only study to date which has developed a risk model framed as a decision support tool. To our knowledge, it is also the only algorithm deployed in practice – during a trial in 2010 in South Korea – where investigators observed a significant reduction in PU incidence from 21 percent to 4 percent.

The majority of previous studies characterizing PUs in the ICU have used manual audits of clinical notes to profile relatively small patient cohorts. For example, the number of PU patients in study cohorts varied between 16 and 140 in audits conducted by numerous studies [18,19,20]. Very limited work has been done to automatically extract PU information using structured EHR data. This is despite the increasingly widespread use of template-based reporting systems for nurse skin examinations, and the fact that EHR-derived PU data has consistently been found to more accurately represent the prevalence of ulcers than clinical progress notes [21,22]. ICUs have the highest rates of hospital acquired ulcers; however, only one study to date has utilized ICU-specific EHR data at scale to characterize the disease burden of pressure injury [15].

To address these gaps in literature, we conducted a study to predict PU in the ICU using EHR structured data. The first aim is a large-scale descriptive study of PUs in an observational ICU dataset. This forms the basis for the second objective – developing a machine learning model to predict PU development. To our knowledge, this is the first predictive model for PUs built on EHR data from an ICU in the US, and will make use of the largest training dataset to date (for comparison, Kaewprag et al. had 590 cases and 7,127 controls) [15]. Specifically, the study aims to predict future PU development using data from the first 24h of ICU admission, as a means to risk stratify patients early in their care.

## Materials and methods

Our study consisted of four key components: (1) identifying a cohort of patients who develop PUs in the ICU; (2) descriptive statistics comparing the PU versus non-PU populations; (3) assessing the prognostic value of the Braden score within the first 24h of admission for future PU development (to benchmark routine practice); and (4) training and evaluating a range of machine learning classifiers to predict future PU development using EHR data from the first 24h of admission. We outline each of these steps below.

### Data

**Figure 1:**
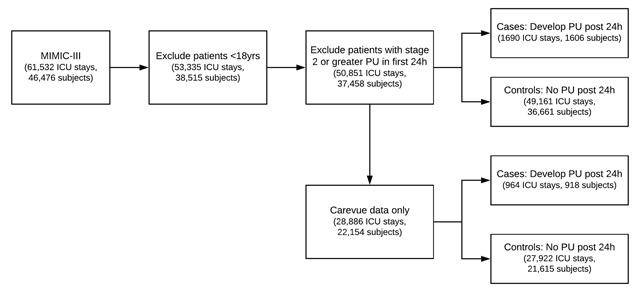
Flow diagram of cohort selection with exclusion criteria.

MIMIC-III (v1.4) is a publicly-accessible database of de-identified EHR data for approximately 54,000 patients admitted to the ICU of Beth Israel Deaconess Medical Center between 2001 and 2012 [23]. The database includes tables for chart events (such as vital signs), medications, diagnosis codes, laboratory measurements, observations and notes recorded by care providers. EHR data are linked to 12-month out-of-hospital survival data obtained from social security records. MIMIC-III spans two EHR recording systems: in 2008 the Beth Israel Deaconess Medical Center switched from the Carevue (Philips Healthcare, Cambridge MA) to the Metavision system (iMDSoft, Wakefield MA). Data were extracted from both systems to create two separate datasets: a collective set of all data, and a Carevue only dataset (since richer data about Braden score were available for these patients). Patients aged under 18 years were excluded (Figure 1).

### Cohort identification and descriptive statistics

PUs were identified using the following ‘ITEMID’ codes for PU staging in the ‘CHARTEVENTS’ table of MIMIC: 551, 552, 553 for Carevue; 224631, 224965, 224966 for Metavision. PUs recorded at stage 2 or above, representing an injury to the dermis, were counted as ulcers. Stage 1 PUs, characterized by redness of the skin with no epidermal breach, were not counted as ulcers as this was deemed to be a highly subjective finding. In total, there were 4,174 ICU admissions where a stage 2 or greater PU was recorded, accounting for 7.8 percent of all admissions. For our prediction task, patients with a PU recorded within the first 24h of ICU admission were excluded (2,001 distinct patients, 2484 ICU stays) because the goal was to risk-stratify patients without a chronic ulcer. Patients who developed a PU after the initial 24h window were classified as cases. This yielded 1,690 cases (1,606 distinct patients) and 49,161 control admissions (36,561 patients). Descriptive statistics were calculated for these populations with each ICU admission counted as a distinct samples. Populations were compared using two sample t-tests for continuous variables and chi-square tests for categorical variables.

### Prognostic evaluation of Braden score

For the Carevue patients, where 93.5 percent of ICU admissions had Braden score documented within 24h, we evaluated the performance of standard Braden score thresholds for high and severe risk (≤12 and ≤9 respectively) in predicting future PU development.

### Feature engineering

**Figure 2:**
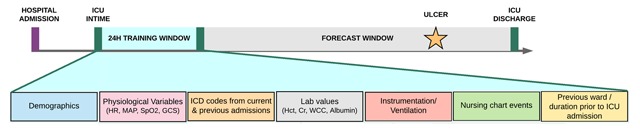
Feature engineering using multiple data sources from the first 24h of ICU admission.

The design of our prediction task is illustrated in Figure 2. Data from the first 24h post ICU admission were used to generate a feature vector in order to predict, as a binary outcome, the occurrence of a PU in the remainder of that admission. A wide range of demographic, clinical, laboratory and environmental features were used to populate the feature vector. The rationale behind feature engineering was to use only common variables that could be readily extracted from the EHR at the 24h timepoint, taking into account known risk factors for PU [24]. Features with greater than 80 percent missingness were not included (consequently, variables such as C-reactive protein were excluded). Braden score data were not used in the original feature vectors, with the intention to determine if EHR-derived model could match or outperform nurse-calculated Braden scores.

A total of 40 features were used to populate the feature vectors (Table 1, excluding the asterisked variables). Demographic features included age, gender and ethnicity. Physiological features included specific vitals (mean arterial pressure (MAP), peripheral oxygen saturation (SpO2) and Glasgow Coma Scale (GCS), which were averaged across the first 24h. Results outside of physiological bounds, as deemed by a critical care physician, were censored. Laboratory features included complete blood counts, electrolytes, albumin, arterial blood gases, blood urea nitrogen, bilirubin, blood glucose and international normalized ratio (INR), with the most recent result within the 24h time window used. The patient’s ventilation status was encoded as: no ventilation, non-invasive ventilation or mechanical ventilation, with the highest level during the first 24h used.

**Table 1:** Demographic and clinical features compared between the PU and non-PU populations. (Features marked with an asterisk were not used as predictor variables.)

	No ulcer (n = 49,161)	Ulcer (n = 1,690)	P value	% missingness

**Demographics**				
Age (years, mean, SD)	63.7 (17.2)	66.5 (15.4)	<0.001	0
Gender (% male)	56.2	60	0.002	0
Weight (kg, mean, SD)	83.6 (25)	87.0 (31)	<0.001	40
Ethnicity (%)			<0.001	0
Asian	2.4	1.6		
Black	9.5	7.5		
Hispanic	3.4	2.7		
Other	2.5	1.7		
Unknown	10.5	12.7		
White	71.6	73.8		
Insurance status (%)			<0.001	0
Public	66.6	71.2		
Private	32.3	28.2		
Uninsured	1.2	0.5		
**Admission**				
Length of stay* (days, median, IQR)	2.1 (3)	11.5 (16)	<0.001	6
Time from hospital to ICU admission (days, median, IQR)	0.03 (1)	0.08 (3)	<0.001	0
No prior admission with ulcer (%)	25.4	33.8	<0.001	0
Admitting ICU ward	NA	NA	<0.001	0
Stage 1 PU in first 24h (%)	1.2	11.1	<0.001	NA
Pressure reduction device	1.2	65	<0.001	NA
Time to ulcer onset* (median, IQR)	NA	45 (7.1)	NA	NA
Ulcer healing* (%)	NA	29.6	NA	NA
Braden score* (median, IQR)	15.0 (4)	13.0 (3)	<0.001	47
Noninvasive ventilation (%)	3.5	3.4	<0.001	0
Mechanical ventilation (%)	39.4	67.9	<0.001	0
Mortality within 12 months* (%)	41.6	55.9	<0.001	0
**Physiology (mean, SD)**				
Glasgow Coma Scale (GCS)	12.3 (13)	9.6 (3.7)	<0.001	45
Mean Arterial Pressure (MAP) (mmHg)	79 (12)	76 (12)	<0.001	44
Oxygen saturation (%)	97 (2)	97 (2)	3	44
Arterial pO_2_ (mmHg)	163 (71)	148 (57)	<0.001	67
Arterial pCO_2_ (mmHg)	41 (9)	41 (10)	0.6	67
Hemoglobin (g/dL)	10.8 (1.8)	10.3 (1.6)	<0.001	6
Hematocrit (%)	31.8 (5)	30.6 (4)	<0.001	37
White blood cell count	11.8 (9)	13.9 (10)	<0.001	11
Neutrophils (%)	78.8 (15)	79.5 (15)	0.45	11
Platelet count	218 (111)	218 (125)	0.9	11
Blood glucose (mg/dL)	138 (50)	148 (57)	<0.001	10
Sodium (mmol/L)	138 (4)	138 (5)	0.1	1
Potassium (mmol/L)	4.1 (0.6)	4.2 (0.6)	0.3	1
Creatinine (mg/dL)	1.4 (1.5)	1.8 (1.6)	<0.001	1
BUN (mg/dl.)	24.4 (20)	34.7 (25)	<0.001	1
Albumin (g/dL)	3.1 (1.2)	2.8 (0.6)	<0.001	77
Total bilirubin (mg/dL)	1.9 (4)	2.6 (6)	0.02	70
Troponin (ng/mL)	1.2 (4)	1.1 (4)	0.7	74
INR	1.5 (1)	1.6 (1)	<0.001	29
**Chronic diseases (%)**				
Diabetes	27	22	<0.001	0
Neuropathy	5	4	0.2	0
Peripheral vascular disease	27	22	<0.001	0
Amputation	0.1	0	0.7	0
Spinal cord injury	0.2	0.1	0.9	0
Coronary artery disease	4	4	0.9	0
Leukemia	1	1	0.5	0
Stroke	1.5	15	0.4	0
Heart failure	27	25	0.03	0
Anemia	18	14	<0.001	0

Comorbidities were evaluated by mapping International Classification of Diseases (ICD) codes from the current and previous admissions to 10 high-level diagnoses: anemia, coronary artery disease, amputation, heart failure, diabetes, leukemia, neuropathy, peripheral vascular disease, spinal cord injury and stroke. These comorbidities were chosen because of known associations with PU development, either due to immobility or impaired wound healing. Our reasoning for using ICD codes from the present admission was that these conditions are all chronic. Even when an ICD is assigned at discharge, it would likely have been present in the problem list of a patient even within the first 24h. If a patient had a previous ICU admission, a recording of stage 2 or greater PU during that admission was encoded as a specific feature. Additional features included the patient’s previous ward location, current ICU ward, and the duration from hospital admission to ICU admission. Features were harmonized across the Metavision and Carevue datasets. The range and missingness of features were evaluated in consultation with clinical mentors, with an iterative process for refining the scope of features selected.

### Classifier training

**Figure 3:**
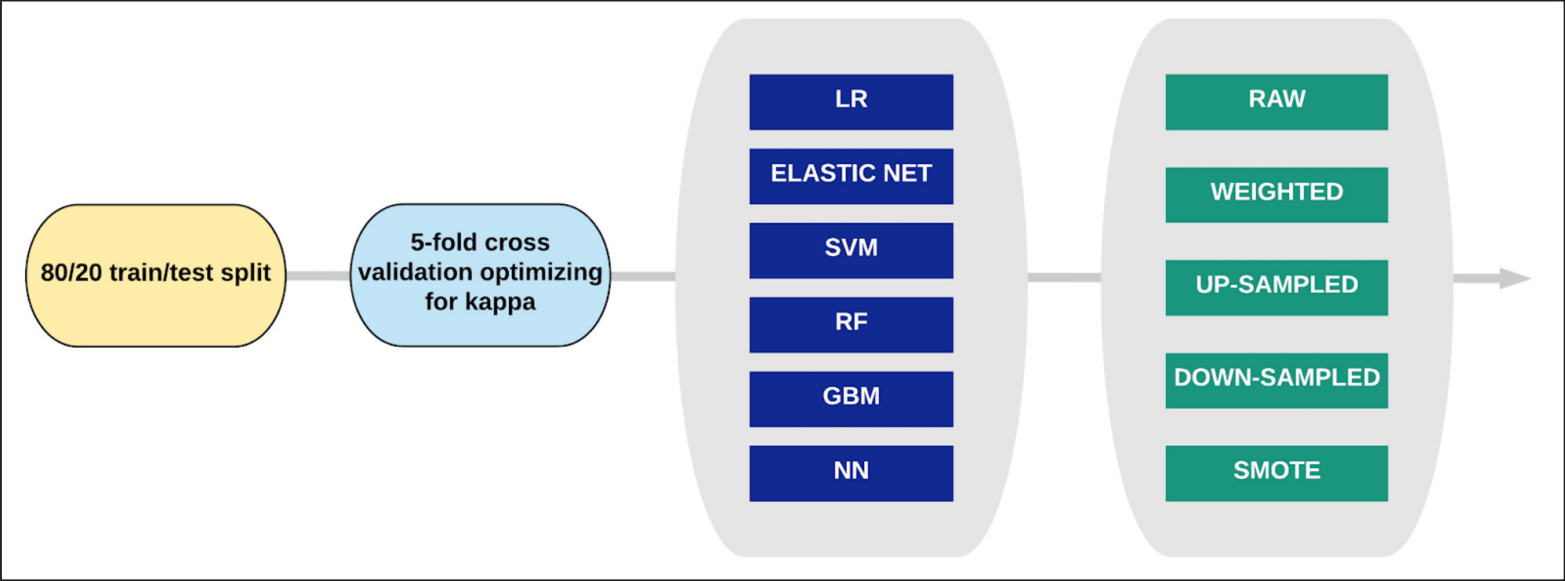
Classifier training pipeline illustrating the range of models and sampling techniques used.

Figure 3 shows the model development pipeline. Models were built using both the entire dataset, and the Carevue data only. We trialed both median imputation and k-nearest neighbors imputation (with 5 nearest neighbors) to populate missing values. Results are reported for median imputation as this method showed superior performance overall. For regression models, categorical variables were dummy coded using full-rank encoding, except for ICD codes which were encoded with one-hot encoding. Continuous variables were standardized.

Data were split into training and test sets with an 80:20 ratio. The following classifiers were trained using 5-fold cross-validation on the training set over a standard search grid of hyper-parameters: logistic regression (LR), elastic net, support vector machine with a linear kernel (SVM), random forest (RF), gradient boosting machine (GBM), and a feed-forward neural network with a single hidden layer. During cross-validation, classifiers were optimized on Co- hen’s kappa score – a measure of variability between the expected and observed accuracy that better accounts for class imbalance (3.3 percent PUs) [25]. Model performance is reported as precision and recall on the test set.

We cross-validated each classifier with a range of minority class sampling techniques including class weightings, up-sampling the minority class, down-sampling the majority class, and Synthetic Minority Oversampling Technique (SMOTE). As above, each classifier was tuned with 5-fold cross validation and the final model was evaluated on the test set.

As a final experiment, the optimal classifier was re-trained using Braden score parameters in addition to the original predictor variables. This included the overall Braden score as well as scores for the six sub-components: mobility, activity, moisture, shear, nutrition and sensory. Cross-validation and test set validation were as described above.

## Results

### Patient characteristics

Table 1 compares a range of demographic and clinical features between the PU (post 24h) and non-PU populations, with each admission counted as distinct. The mean weight and age were higher among patients with PUs, as was the median length of stay. Additionally, PU patients had higher rates of ventilatory support (both non-invasive and mechanical ventilation), lower mean arterial pressure and lower arterial oxygen pressure. Of these 1690 PU patients, the median time to onset was 4.2 days post ICU admission and 29.6 percent demonstrated healing during the ICU, as evidenced by a reduction in stage between the final-recorded stage and maximum stage.

### Prognostic performance of Braden score

**Figure 4:**
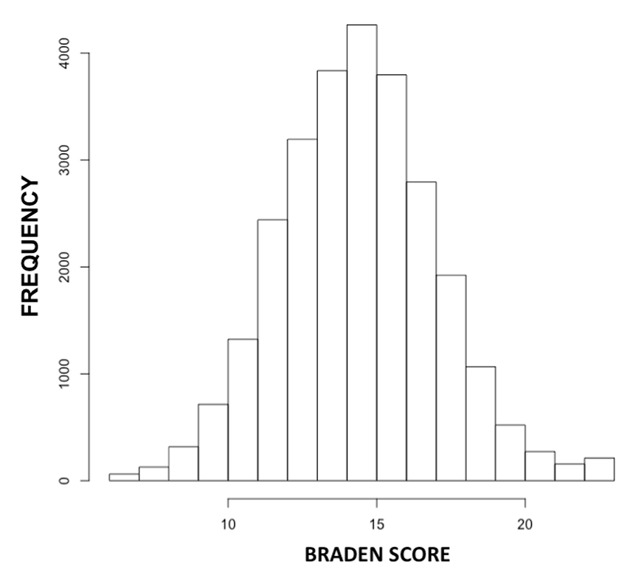
Histogram of Braden scores measured within the first 24h of admission for Carevue patients (range 6–23).

**Figure 5:**
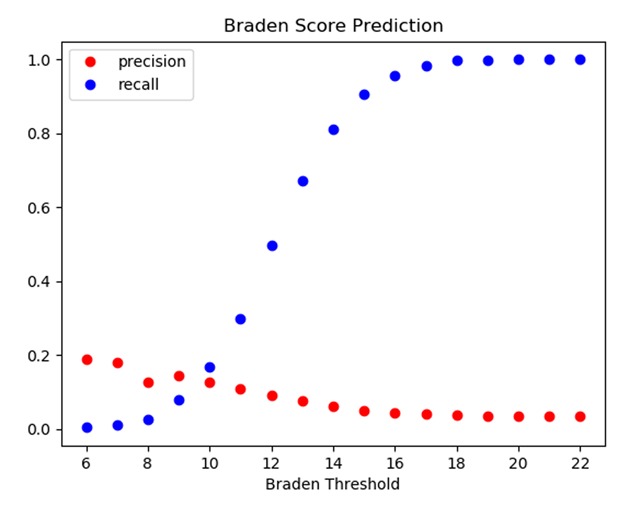
Precision and recall at various thresholds of Braden score (measured within 24h) in predicting future PU development.

Figure 4 shows the distribution of Braden scores at 24h across the Carevue population. Figure 5 shows the precision and recall of various thresholds of the Braden score at 24h in predicting future PU development. High risk is defined as a score of 12 or below and severe risk as 9 or below [26,27]. The high-risk threshold had precision 0.09 and recall 0.50; while the severe-risk threshold had precision 0.15 and recall 0.08.

### Classifier performance

The optimal classifier on the entire dataset was a logistic regression model, which also had precision 0.09 and recall 0.71. The optimal classifier on the Carevue-only dataset was a single layer neural network with model weights, which showed precision 0.09 and recall 0.70 on the test set; followed by a weighted logistic regression with precision 0.09, recall 0.67.

The performance of tuned models on the test set is shown in Table 2. Many of the models operated as majority classifiers in the unweighted scenario, labeling every case as a non-ulcer. Kappa score, and performance of the test set, tended to increase when trained with model weights or SMOTE sampling.

**Table 2:** Classifier performance on Carevue-only and all data, based on raw training data (unweighted), model class weights (weighted) or synthetic minority oversampling (SMOTE).

Carevue (n = 28,886)						

	Unweighted		Weighted		SMOTE	

	Precision	Recall	Precision	Recall	Precision	Recall

Braden Score	0.09	0.5				
LR	0.1	0.005	0.09	0.67	0.12	0.49
Elastic Net	0.11	0.005	0.09	0.67	0.13	0.48
SVM	NA	0	NA	0	0.12	0.48
RF	0.33	0.02	0.3	0.02	0.18	0.17
GBM	0.11	0.18	0.04	0.91	0.16	0.36
Neural net	0.33	0.01	0.09	0.7	0.11	0.52
**All Data (n = 50,851)**						

LR	0.67	0.006	0.09	0.71	0.12	0.46
Elastic Net	0.67	0.006	0.09	0.7	0.12	0.46
SVM	NA	0	NA	0	0.12	0.44
RF	NA	0	0.33	0.006	0.18	0.28
GBM	0.11	0.18	0.04	0.94	0.17	0.38
Neural net	0.06	0.006	0.09	0.7	0.11	0.49

### Feature importances

Table 3 shows the standardized regression coefficients of the top 10 predictors in two of the highest performing models, ranked based on the absolute value of the t-statistic for each model parameter with the standardized regression coefficient shown. Multiple features are represented in both, including stage 1 PU, GCS, blood urea nitrogen (BUN), paO_2_, and albumin.

**Table 3:** Top 10 features for the weighted logistic regression models trained on Carevue data (left) and on all data (right) based on relative variable importance.

Weighted Logistic Regression	Carevue	All data	

Variable	Standardized coeff.	Variable	Standardized coeff.

Stage 1 PU within first 24h	2.0	GCS	–0.65
GCS	–0.46	Stage 1 PU within first 24h	2.1
BUN	0.28	BUN	0.4
paO_2_	–0.36	paO_2_	–0.37
Cardiac Surg. Recovery Unit	–0.79	Cardiac Surg. Recovery Unit	–0.88
Albumin	–0.39	Albumin	–0.41
Medical ICU	–0.59	Hemoglobin	–0.54
Pressure reduction device	0.89	Medical ICU	–0.53
Mechanical ventilation	0.61	Pressure reduction device	1.1
Mean arterial pressure	–0.19	Mean arterial pressure	–0.17

### Classifier with Braden

When Braden features were integrated into the feature matrix, the optimal classifier on Carevue data, the weighted logistic regression, showed essentially unchanged performance relative to the classifier without Braden data – precision 0.09, recall 0.68.

## Discussion

In this large EHR-based study, we demonstrate that a weighted logistic regression using 40 EHR-derived features from the first 24h of an ICU admission outperformed the nurse-calculated Braden score in recall and matched its precision. Given the recall boost, this type of EHR-based classifier may have utility as an automated screening tool for PUs early in a patient’s admission. Preventing a PU in a high-risk patient is more effective and less costly than treating an evolving PU. Moreover, interventions such as frequent rolling and foam padding are relatively low-cost, increasing our tolerance for false positives. This work provides a clear example of how routinely collected EHR data can inform the design of care protocols.

The logistic regression model is interpretable and allows clinicians to better understand the impact of demographic and physiological factors on PU development. Important features were broadly consistent with domain knowledge and clinical intuition surrounding PUs. For example, it is intuitive that mechanical ventilation, low GCS, low albumin, and oxygen saturation would be positively associated with PU development as they are proxies for immobility, impaired nutrition, and poor wound healing. Intuitively, the observation of a stage 1 PU within the first 24h was a strong predictor of future development of more severe PUs. The time between hospital admission and ICU admission was also positively correlated, supporting the hypothesis that longer patient stays on the wards prior to ICU admission are associated with PU incidence. Interestingly, the model identified that admission to certain units within the ICU (e.g. medical versus surgical) was associated with downstream PU incidence, highlighting high-performing units whose pressure care protocols might be emulated.

Including Braden features in the model did not improve performance, suggesting that the Braden scoring system does not add significant information to the EHR-based algorithm. As Kaewperg et al. describe, Braden subscales for activity, nutrition, mobility, sensory perception, and moisture are often not useful predictors because they tend to have similar values among ICU patients [15]. By incorporating a broader range of EHR-derived features, our model can be used to capture risk factors that meaningfully differ between patients with critical illness, and thereby achieve better discrimination of risk status. Additionally, an EHR-based model removes the need for nurses to manually calculate Braden risk scores, which is time consuming, subjective, and can easily be overlooked.

The descriptive statistics provide a unique insight into the burden of PUs in a tertiary ICU, using EHR data alone. The prevalence of PUs has been quoted as high as 49 percent, however we find 7.8 percent of admissions have a PU of stage 2 or higher. Of the 1,690 PU cases that developed after 24h (which removes chronic PUs), 29.6 percent demonstrate healing during the patient’s admission (defined as a final PU stage less than the maximum recorded stage), which is a metric not previously investigated in EHR data.

Our study is subject to some limitations. First, the underlying pathophysiology of PUs may simply not be reflected in EHR data, making it a particularly challenging prediction task. The main driver of PUs is consistent skin pressure over bony prominences, and although we can find proxies in the EHR for immobility and impaired healing, these are surrogate features that do not directly capture the pressure waveform on, for example, a patient’s sacrum [24]. This makes it very challenging to predict future onset of pressure injury. Second, the EHR features that are available are affected by missingness. For example, a significant proportion of the population are missing height and weight data, preventing the calculation of Body Mass Index (BMI), an important risk factor in PU development. Third, there was a significant class imbalance in our study design, with only 3.3 percent of cases developing a PU. Many of the classifiers defaulted to predicting the majority class. This was addressed with selection of kappa score as the optimization metric, along with several class re-balancing techniques; however we only observed incremental performance improvements. Fourth, our study was based on a large, academic tertiary medical center, and may not be representative of smaller community facilities. Fifth, our cohort ended in 2012, and important trends in public health have occurred in the interval. The prevalence of obesity, a major risk factor for PUs, did not increase in the overall US population during the time period of data collection but has increased both in the US and in other countries in the intervening period [28,29]. While this can affect model performance, it also makes PU prevention increasingly important. In the same vein, new treatments for PUs have been introduced in the interim, for example foam mattresses with a range of pressure settings and alternating-pressure overlays [30].

Future work will involve an iterative process of feature engineering and model tuning to increase the precision of our classifier. A higher precision model could help to inform the allocation of expensive prophylactic resources such as pressure support mattresses. Additionally, the model must be deployed as a clinical decision support tool, as in Cho et al., to fully evaluate its impact on PU incidence. This is the practical benchmark for utility, beyond precision and recall scores [11]. Although this experiment had significant clinician input throughout model design (including from informaticians, practicing intensivists and nursing staff); its safety and clinical utility must be assessed before translation into a decision support tool. Future work should also include assessing the extensibility of the model to other centers, given the ubiquity of EHRs and the commonplace use of the covariates in this model.

## Conclusion

In this paper, we develop a model for predicting future PU development at 24h, which outperforms the commonly used, resource-intensive Braden scale. This model uses EHR data elements and could be a means to automatically screen patients for PU risk early in an ICU admission, either as an adjunct to, or substitute for, repeated manual Braden scoring by nurses. The optimal models show an association between PU development and EHR-derived proxies for immobility (e.g. spinal cord injury and low GCS), nutritional status (e.g. low albumin) and impaired skin healing, (e.g. low paO_2_). While additional refinement of our model is warranted, implementation of this kind of EHR-based model as a decision support tool may decrease the incidence of PUs, as has been demonstrated in other literature. Data-driven risk stratification may be a means to inform resource allocation and improve quality across ICUs internationally.
